# Supplementary figures and images for: Anti-epileptic drug topiramate upregulates TGFβ1 and SOX9 expression in primary embryonic palatal mesenchyme cells: Implications for teratogenicity
Source: PLoS One. 2021 Feb 12;16(2):e0246989. doi: 10.1371/journal.pone.0246989 (PMC7880431; doi:10.1371/journal.pone.0246989)

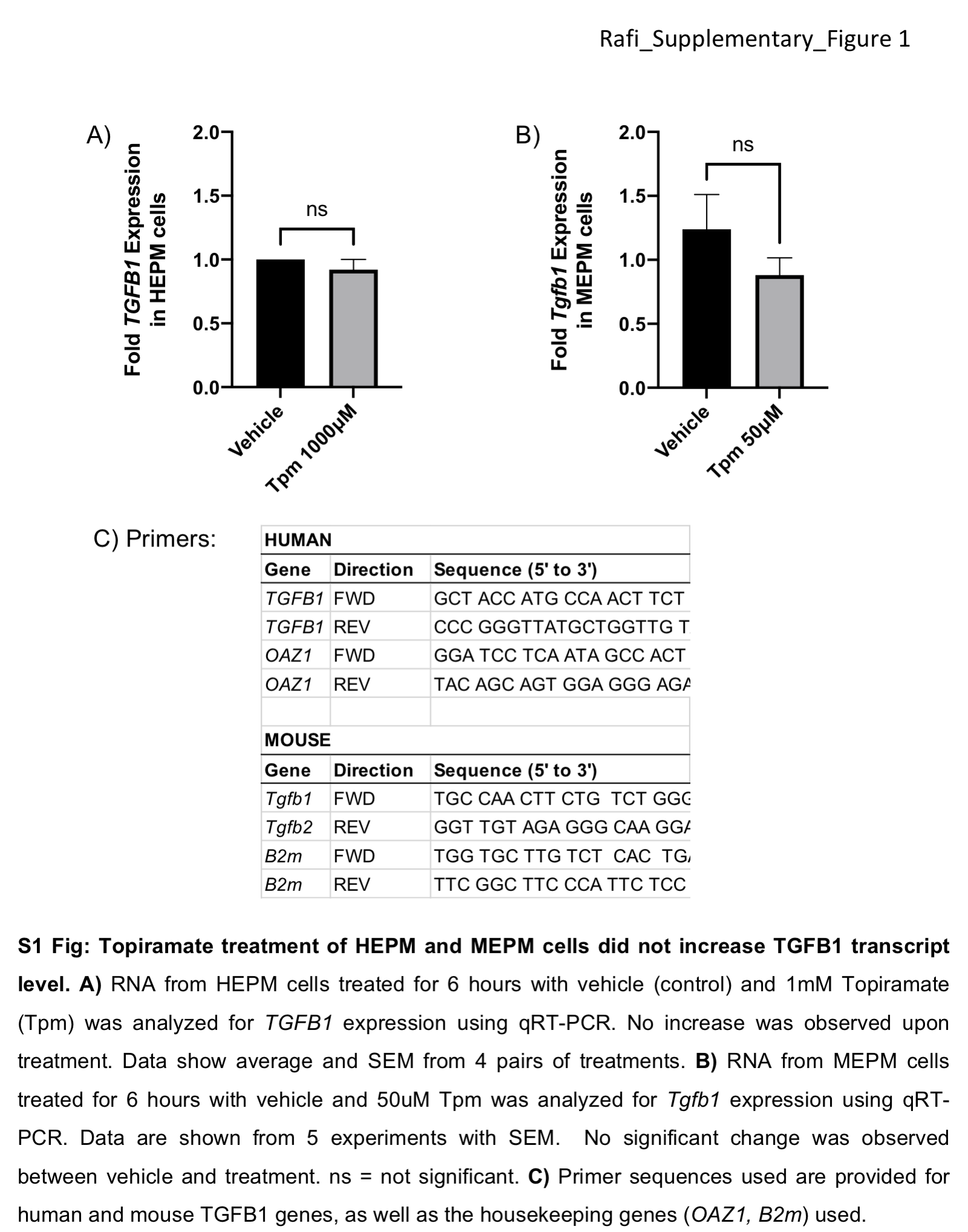

Supplement: S1 Fig — A) RNA from HEPM cells treated for 6 hours with vehicle (control) and 1mM Topiramate (Tpm) was analyzed for TGFB1 expression using qRT-PCR. No increase was observed upon treatment. Data show average and SEM from 4 pairs of treatments. B) RNA from MEPM cells treated for 6 hours with vehicle and 50uM Tpm was analyzed for Tgfb1 expression using qRT-PCR. Data are shown from 5 experiments. No significant change was observed between vehicle and treatment. ns = not significant. (TIF) [file pone.0246989.s001.tif]

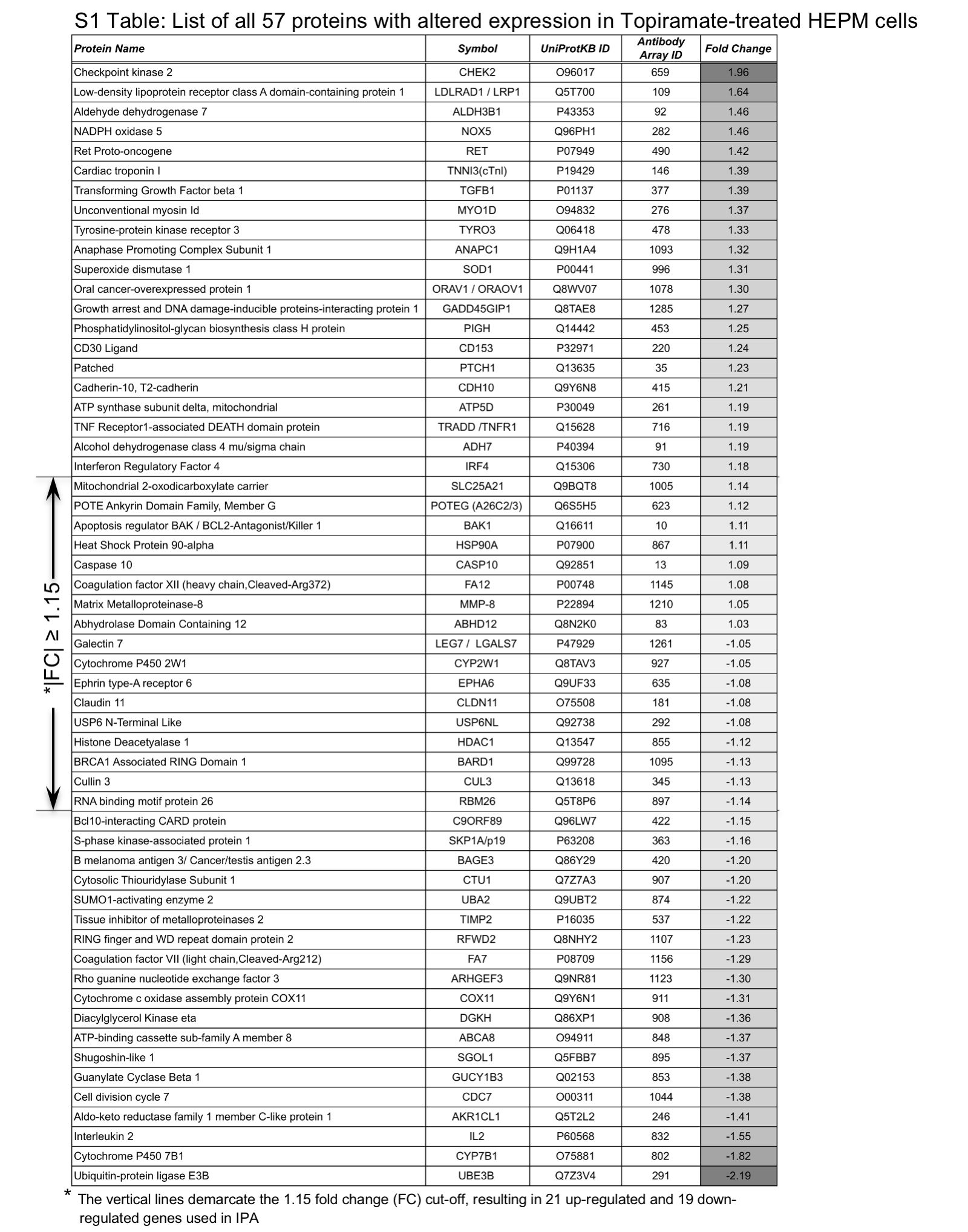

Supplement: S1 Table — (TIF) [file pone.0246989.s002.tif]

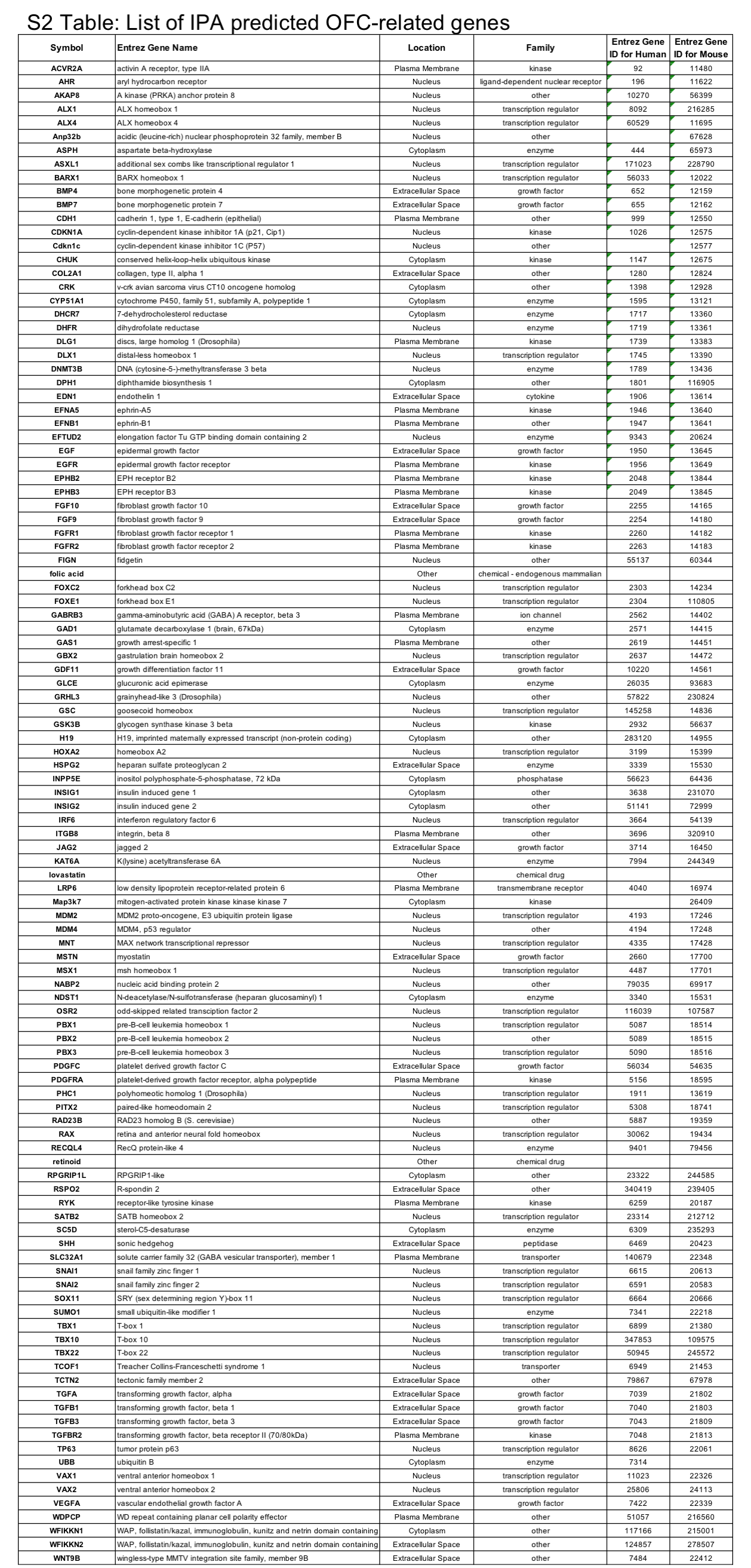

Supplement: S2 Table — (TIF) [file pone.0246989.s003.tif]

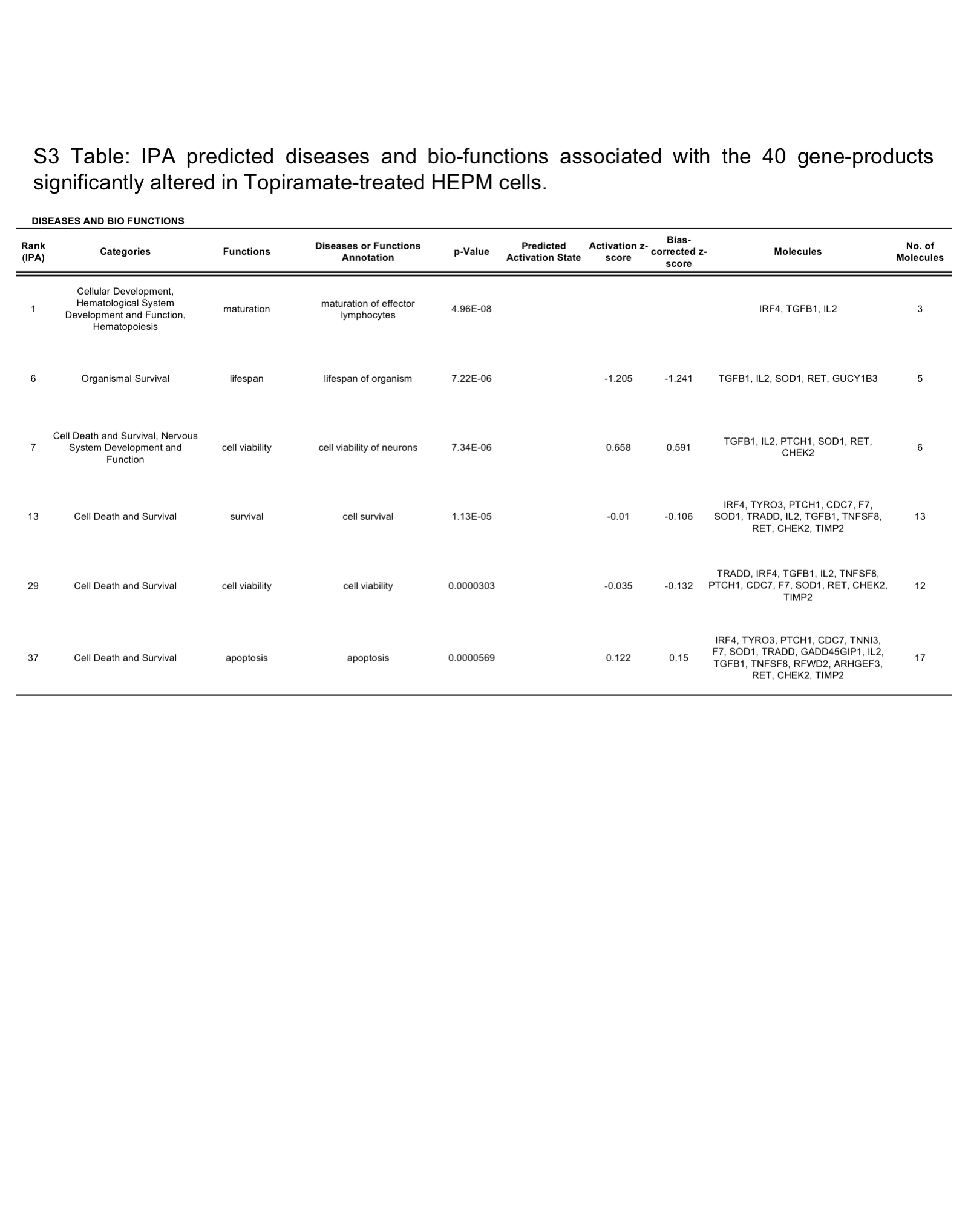

Supplement: S3 Table — (TIF) [file pone.0246989.s004.tif]

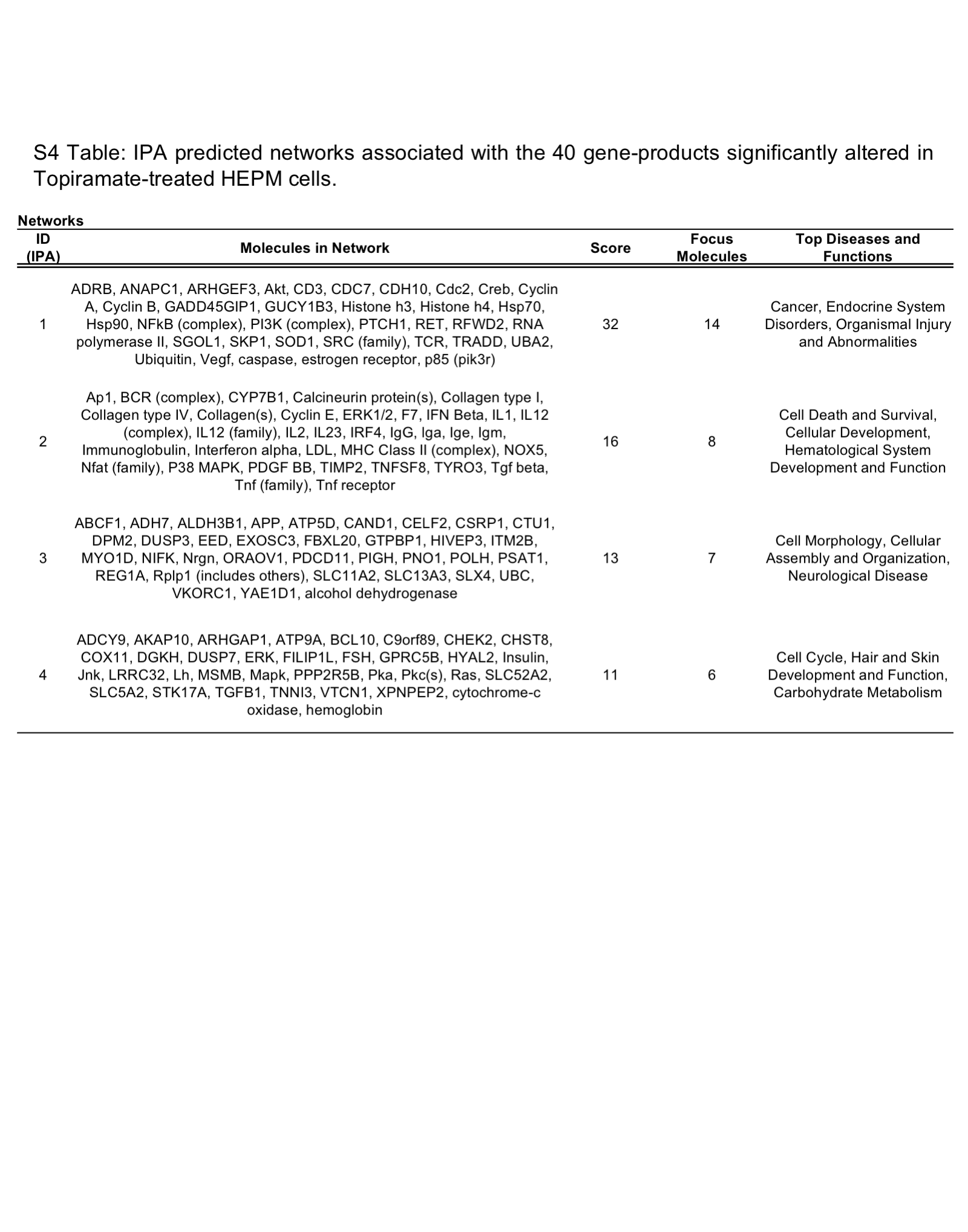

Supplement: S4 Table — (TIF) [file pone.0246989.s005.tif]
