# Supplementary material for: Anti-epileptic drug topiramate upregulates TGFβ1 and SOX9 expression in primary embryonic palatal mesenchyme cells: Implications for teratogenicity
Source: PLoS One. 2021 Feb 12;16(2):e0246989. doi: 10.1371/journal.pone.0246989 (PMC7880431; doi:10.1371/journal.pone.0246989)

S1 Raw Images

Figure 2F (TGFβ1)

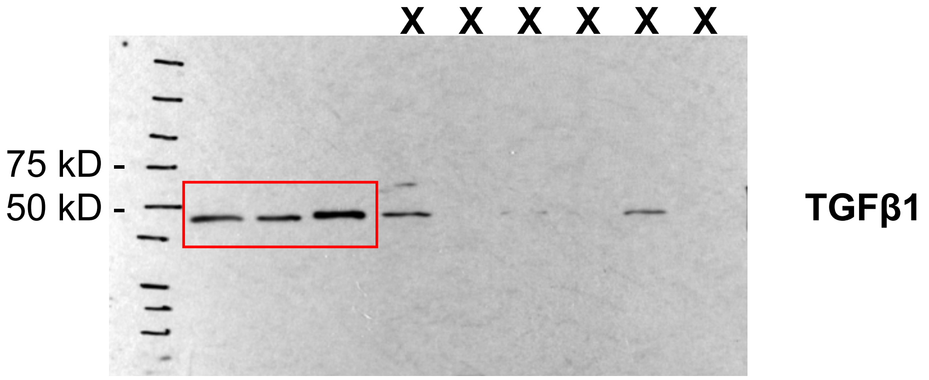

Figure 2F (TGFβ1 – total protein loading)

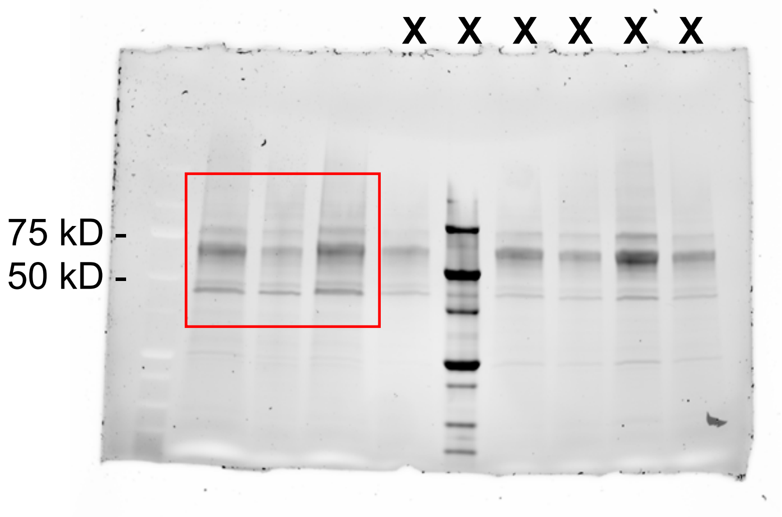

Figure 2F (P-SMAD2)

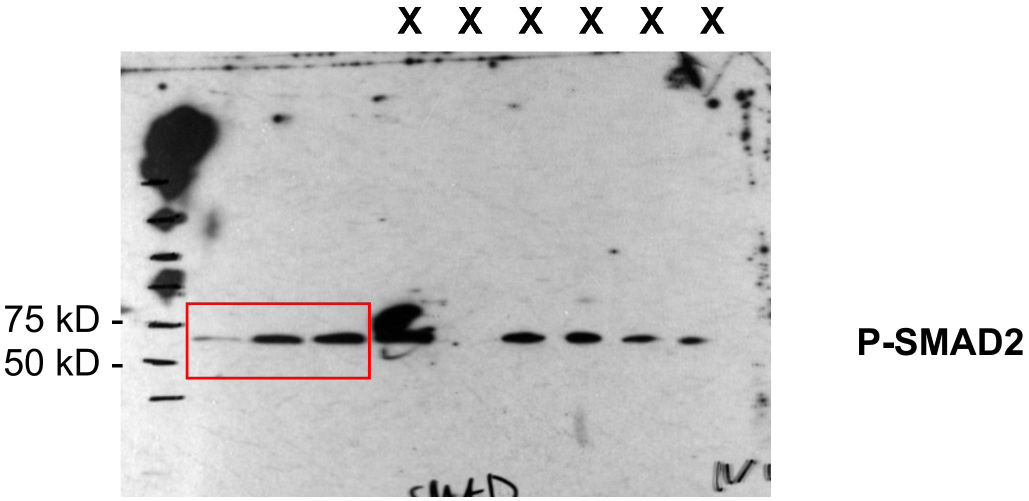

Figure 2F (P-SMAD2 – total protein loading)

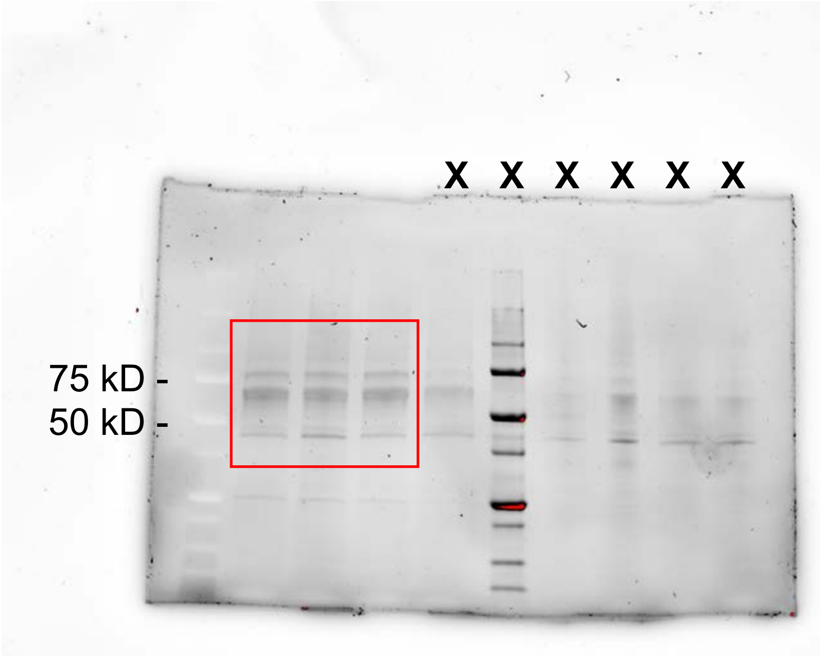

Figure 2F (SMAD2)

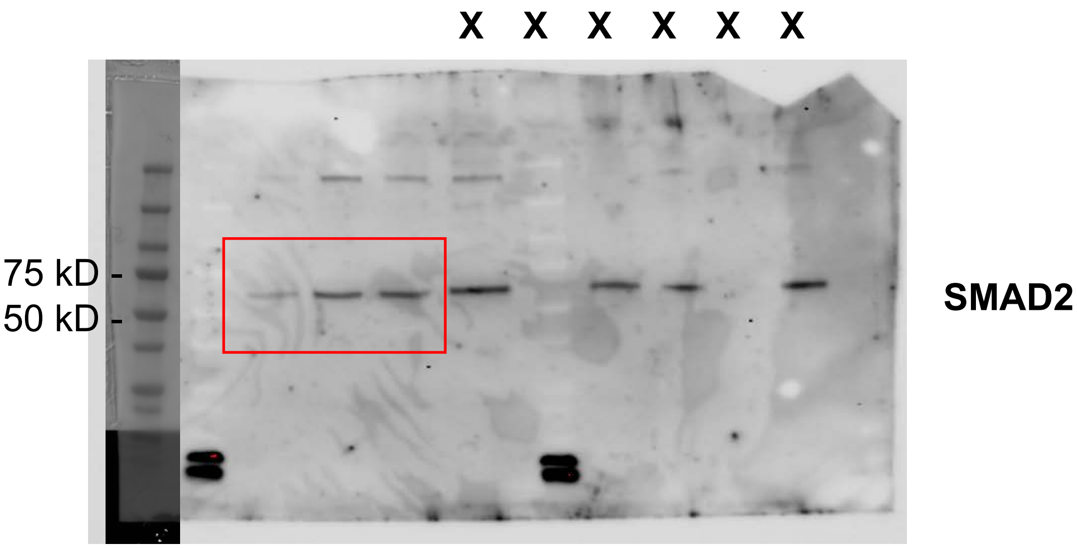

Figure 2F (SMAD2 – total protein loading)

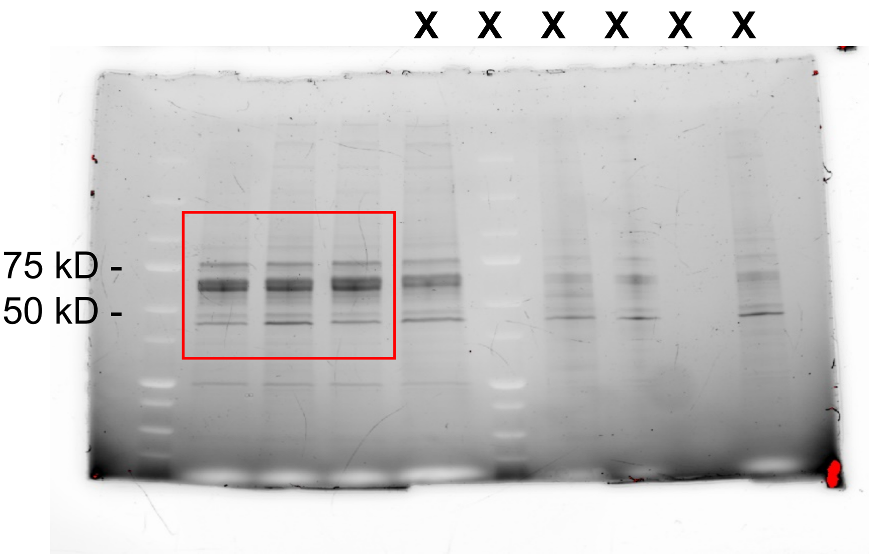

Figure 2F (TGFβ1 – Flumazenil treatment)

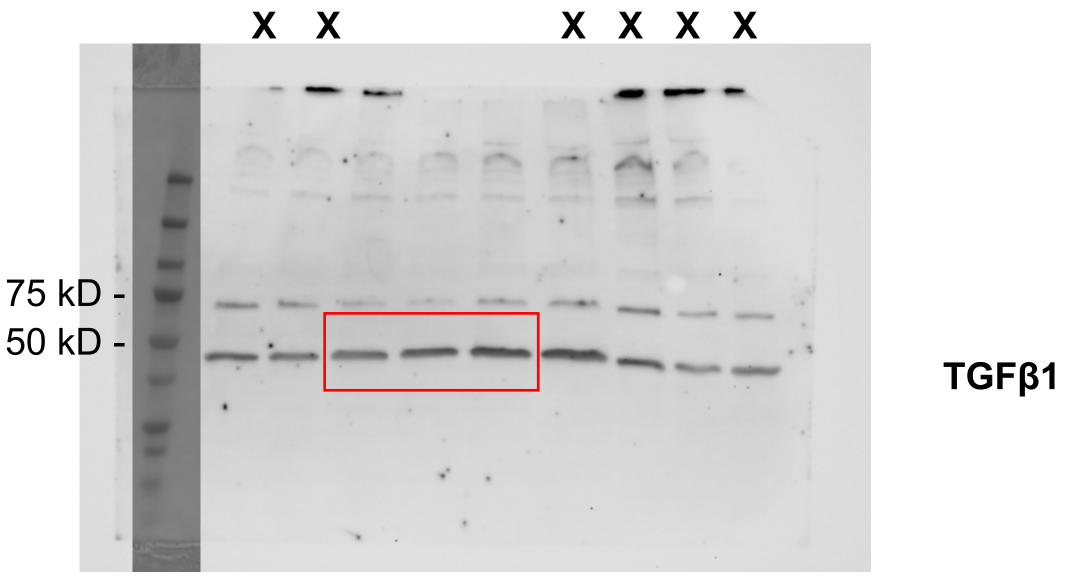

Figure 2F (TGFβ1 – Flumazenil treatment – total protein loading)

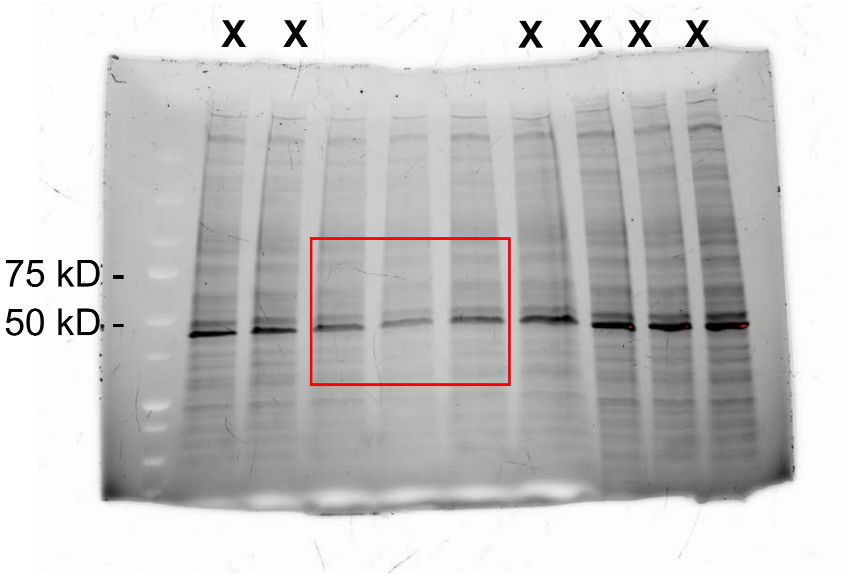

Figure 3A (SOX9)

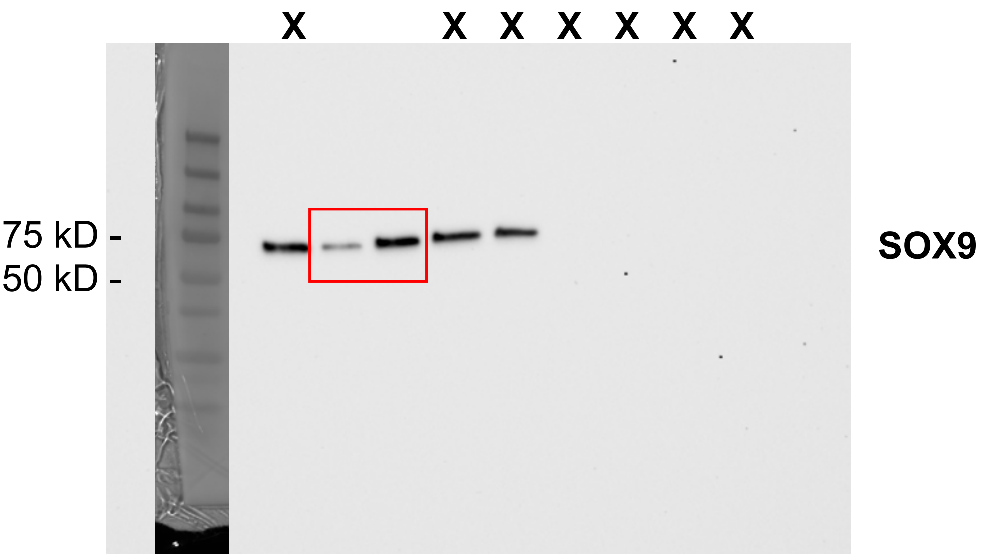

Figure 3A (SOX9 – total protein loading)

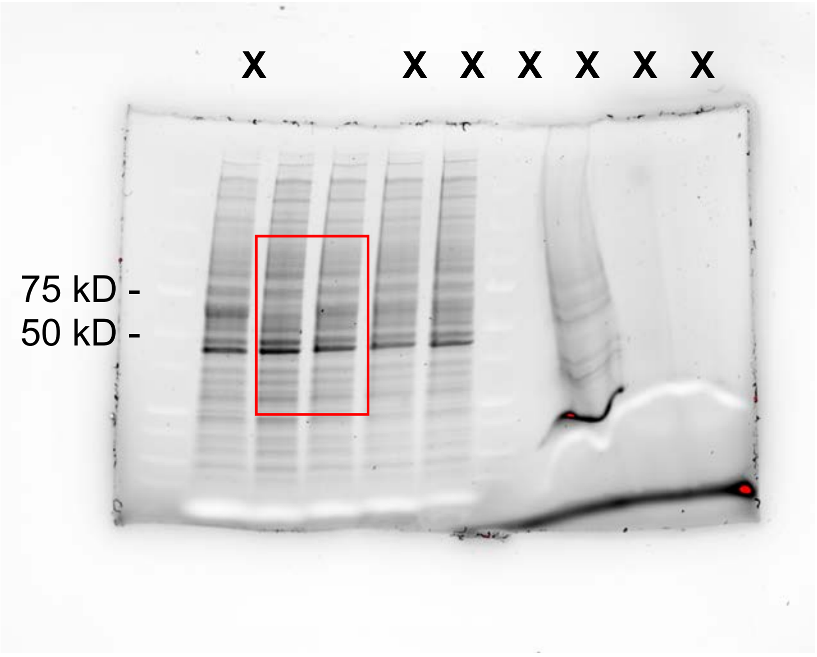

Figure 3A (SOX10)

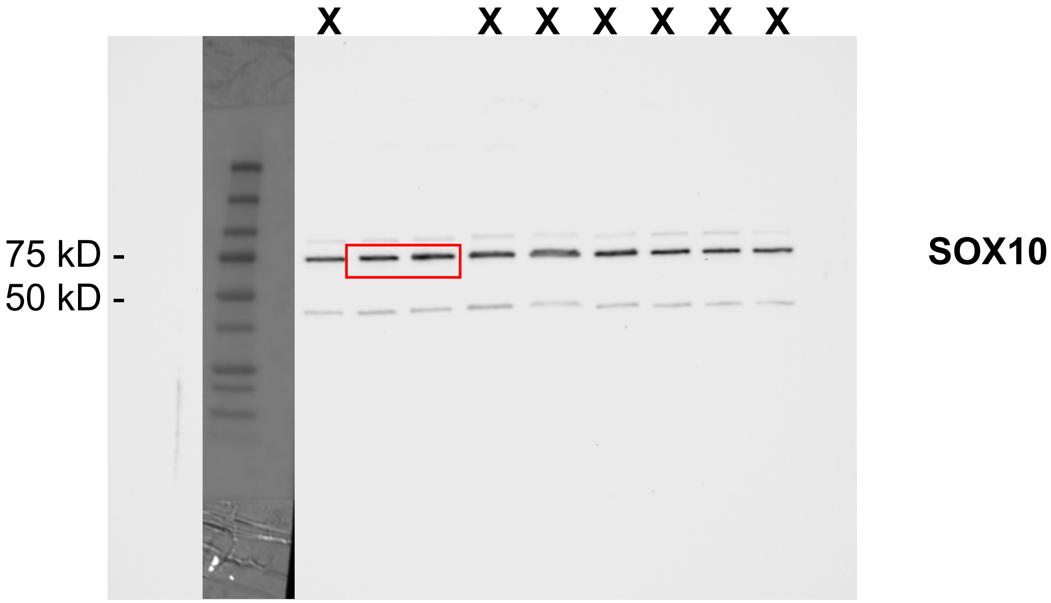

Figure 3A (SOX10 – total protein loading)

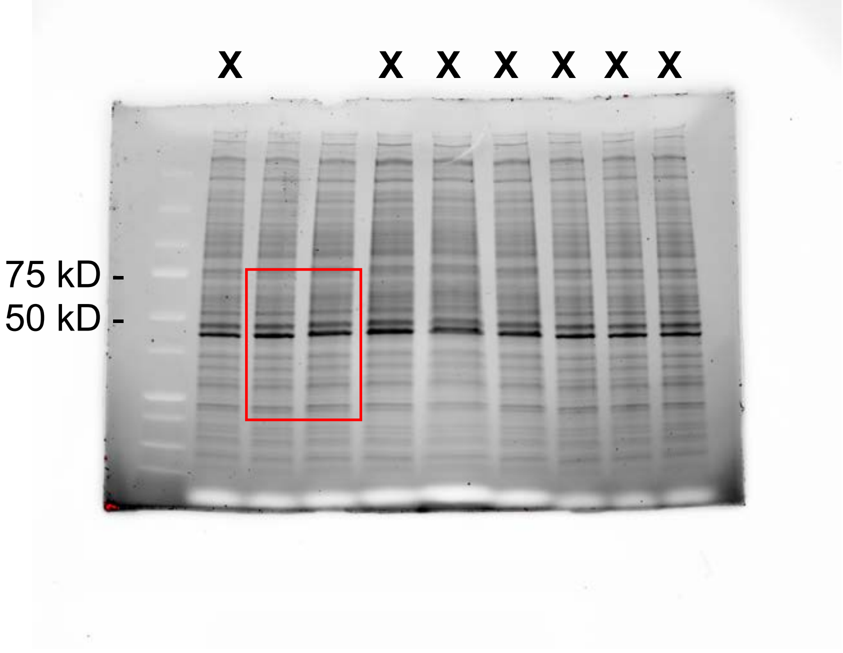

Supplement: S1 Raw images — (PDF) [file pone.0246989.s006.pdf]
