# Supplementary material for: Anti-epileptic drug topiramate upregulates TGFβ1 and SOX9 expression in primary embryonic palatal mesenchyme cells: Implications for teratogenicity
Source: PLoS One. 2021 Feb 12;16(2):e0246989. doi: 10.1371/journal.pone.0246989 (PMC7880431; doi:10.1371/journal.pone.0246989)
Supplement: Attachment — Submitted filename: Topiromate TGF.pdf [file pone.0246989.s007.pdf]

Topiromate TGF-beta review

Exposure to Topiromate in utero is associated with increased incidence of cleft palate among other craniofacial defects however the mechanism by which topiromate affects craniofacial development has not been understood. Here, Rafi et al. use an antibody array to identify changes in protein concentrations in primary palate cultures that are exposed to Topiromate. TGF-beta1 is identified as an overexpressed protein with exposure to topiromate. The authors show topiromate increases phosphorylation of Smad2, a readout of TGF-beta signaling. They also show that TGF-beta target gene (Sox9) expression is increased upon topiromate exposure. This work is important because topiromate is a commonly used medication to treat epilepsy, migraine, and bi-polar disorder. It is important that clinicians and patients understand the risks associated with the medication and the mechanism by which the medication may disrupt development.

Suggestions:

Topiromate is also used as a migraine preventative medication. Because migraines are more common than epilepsy, it is worth referencing this in both the abstract and introduction. Migraine affects almost a third of the female population who are of child bearing age, so this is also really important for the significance of this study.

Add a transition between the sentence "Despite broad use and 75 teratogenic potential of topiramate, the molecular mechanism(s) underlying the increased76 occurrence of major congenital malformations is not understood (6, 7, 10, 12, 16)." And the next paragraph.

(SOX9), "a known 92 clefting gene and TGFB1 target."- this phrase is a little unclear. It would be more clear to say "a TGFB1 target that causes causes cleft palate when overexpressed"- or something that states overexpression causes cleft palate rather than loss of function.

Please refrain from using acronyms when possible, for example "OFC" could be replaced with "gene associated with oral or facial clefts. This would make the text more accessible to the general readership of this journal.

Cell cycle and apoptosis genes were also identified in the IPA. Increased apoptosis and decreased cell proliferation could also cause clefting. The focus on TGF-beta is great, but the authors also could have focused on a direct mechanism by looking at cell proliferation and apoptosis.

This is interesting: "We saw a significant increase in intracellular TGFb1 level at 246 the physiological 50μM topiramate treatment (Figure 2E)." We don't expect TGFb1 to function intracellularly. Was there not an increase in TGFb1 in the media, extracellular

portion? Or did the authors not check? IF the authors have not checked, I suggest checking and comparing TGF-beta in the media as well.

Figure 2A-E: The representative cells for 250 uM topiromate show a clear increase in fluorescence. However, D, 1mM topiromate looks like it has less fluorescence than C or B. If there is indeed a greater fluorescence in most cells treated with 1mM topiromate, please find a different representative image. The apparent discrepancy between fluorescence in figure 2D and the quantification in E made me wonder about how the quantification of fluorescence was measured. There are no details about whether the fluorescence is quantified and normalized to the number of cells or if each individual cell is outlined as a region of interest. If the fluorescence is not normalized to the number of cells in an image in any way, the fluorescence quantification is questionable. These details should be provided to allow the reader to interpret the analysis.

I would be interested to see if the upregulation of TGF-beta1 is due to an increase in transcription of TGF-beta1 on the mRNA level or only at the level of protein. Please perform Q-RT PCR to determine if mRNA levels of the ligand are upregulated

Were P-Smad2/3 levels from the nuclear fraction or total cell lysates? Also, were Smad2 protein levels also quantified? It would be interesting to know if Smads are also upregulated or if only the phosphorylation of Smad2/3 are increased with the topiromate treatment.

Consider changing color scheme from Red and Green in Figure 4 to colors that are color-blind friendly.
