# Supplementary material for: Anti-epileptic drug topiramate upregulates TGFβ1 and SOX9 expression in primary embryonic palatal mesenchyme cells: Implications for teratogenicity
Source: PLoS One. 2021 Feb 12;16(2):e0246989. doi: 10.1371/journal.pone.0246989 (PMC7880431; doi:10.1371/journal.pone.0246989)
Supplement: Attachment — Submitted filename: Response to Reviewers.pdf [file pone.0246989.s008.pdf]

We would like to thank both the reviewers for their insightful comments. We have done our best to address each concern and to incorporate their suggested changes, which have improved this revised manuscript.

Reviewer #1: *This article describes an antibody screen to find downstream targets of topiramate in palatial mesenchymal cells that may be causative in palatial clefting. The manuscript is well written and easy to follow. The procedures and statistics all seem to have been performed appropriately, and the results are interesting, as far as they go. It's just that the study doesn't go very far. It starts out strong by identifying target genes and pathways, but then it stops before doing anything with the results beyond bioinformatics and speculative model building.*

1a) For example, the authors state, " To validate that TGFb1 increase plays a physiological role in the etiology of OFC, we utilized primary MEPM cells." But, that's not really what they did. All they did was look at gene and protein increases in related cells. The same thing with SOX9, they just showed up regulation and Smad phosphorylation. This is a good start, but why not do an experiment to show a change in the physiology of the cells?

1b) They have both a plate mesenchyme cell line and primary palate cells. Both are easy to culture, so why not look for things like changes in proliferation or apoptosis as a consequence of topiramate? The pathway analysis they show predicts that topiramate action through those genes changes cell survival and growth (e.g: see Table S3). Then they could do straight forward knockdown or inhibitor experiments to change Tgfb1 activity or SOX9 protein levels during topiramate treatment.

1c) These simple experiments would go a long way toward validating a potential physiological role for these pathways in topiramate action in vivo. It's fairly easy to draw line diagrams of pathways and build models on paper, but which potential mechanisms are really important to the biology being studied? In other words, how important is Tgfb1 really to the teratogenic effect of topiramate compared to all the other possibilities?

- We agree with the reviewer that these are all valid experiments to further characterize the effect of Topiramate on primary cells. However, we posit that the next advance needed is to take these analyses *in vivo* in an animal system and test these mechanistic possibilities. Unfortunately, *in vivo* studies are beyond the scope of this manuscript. However, we want to publish our findings so that other labs interested in TGF-beta signaling or studying teratogenic effects *in vivo*, can extend our findings.

2) The same principle is true with the speculation about GABA involvement in activating Tgfb1 expression. It's easy to speculate, but it should also be easy to test. Why not do cell culture experiments with and without GABA inhibitor to see if it changes Tgfb1 levels?

- We agree with the reviewer that this is an important aspect of our model. We had planned these experiments previously and have now included the finding (Fig.2) that a selective $GABA_A$ receptor inhibitor (Flumazenil) does indeed blunt the increase in TGFB1 level upon Topiramate treatment.

3) On another subject, I am not clear about why SOX10 expression is a useful control for the specificity of the SOX9 upregulation. Is there some scenario of generalized transcriptional upregulation that involves SOX10 such that it needs to be controlled for and this gene is a good indicator or that? Are SOX9 and 10 genes regulated together in other systems such that this connection needs to be ruled out here?

- SOX9 and SOX10 both belong to the same subgroup (SOXE). Deficiency in both leads to cleft palate, and both can be downstream of TGFB signaling. SOX10 plays a major role in specification of migratory neural crest cells, which give rise to MEPM. We did not use SOX10 as a control, instead, we wanted to test two relevant downstream targets of TGF-beta. In some cell types, expression of SOX9 and SOX10 can be antagonistic, particularly cartilage vs. bone specification. When we saw an increase in SOX9, we wondered if SOX10 would be downregulated, which would be another plausible etiology of cleft palate. However, there was no change in SOX10 expression.

4) I have one final general statement about the organization of the manuscript relating to concision. It seems to me that the Discussion section spends a lot of words restating the results from the previous section, such that they almost seem to blend together. I would recommend that the authors spend some time rewriting the Discussion to be more separate from the Results, more concise, and a little less rambling. For example, the second paragraph of the Discussion goes on for several lines about the three Tgfbeta ligands, their expression in palate, and their possible effects on proliferation of palatal cells. This could really be replaced by a couple of sentences about Tgfb1 and what specifically is known about its effect in palate. That is really what the reader needs to evaluate the importance of the results presented in this study.

- As requested, we have made edits to the discussion, especially to the paragraph#2 given as an example. In particular, to simplify, we now focus on upregulation of TGF-beta signaling. However, we still believe that the reader would benefit from considering overall TGF-beta signaling, not just TGFB1.

Reviewer #2: *Topiramate TGF-beta review*
Exposure to Topiramate in utero is associated with increased incidence of cleft palate among other craniofacial defects however the mechanism by which topiramate affects craniofacial development has not been understood. Here, Rafi et al. use an antibody array to identify changes in protein concentrations in primary palate cultures that are exposed to Topiramate. TGF-beta1 is identified as an overexpressed protein with exposure to topiramate. The authors show topiramate increases phosphorylation of Smad2, a readout of TGF-beta signaling. They also show that TGF-beta target gene

(Sox9) expression is increased upon topiramate exposure. This work is important because topiramate is a commonly used medication to treat epilepsy, migraine, and bi-polar disorder. It is important that clinicians and patients understand the risks associated with the medication and the mechanism by which the medication may disrupt development.

Suggestions:
Topiramate is also used as a migraine preventative medication. Because migraines are more common than epilepsy, it is worth referencing this in both the abstract and introduction. Migraine affects almost a third of the female population who are of child bearing age, so this is also really important for the significance of this study.

- We agree with the reviewer that this is an important point. We have amended the abstract to: "Topiramate is an anti-epileptic drug that is commonly prescribed not just to prevent seizures but also migraine headaches, with over 8 million prescriptions dispensed annually."
- We also included "migraines" in the list of indications in the Introduction, and included the sentence: "Importantly, in a recent study (9), twice as many women with migraines (46%) were prescribed topiramate than women with epilepsy or seizures (20%), exposing an even larger proportion of women of childbearing age."

Add a transition between the sentence "Despite broad use and 75 teratogenic potential of topiramate, the molecular mechanism(s) underlying the increased76 occurrence of major congenital malformations is not understood (6, 7, 10, 12, 16)." And the next paragraph.

- We added the following transition sentence to the next paragraph: "We sought to identify molecular clues to the teratogenic effect of topiramate on palate development."

(SOX9), "a known 92 clefting gene and TGFB1 target."- this phrase is a little unclear. It would be more clear to say "a TGFB1 target that causes causes cleft palate when overexpressed"- or something that states overexpression causes cleft palate rather than loss of function.

- We changed the text to "Topiramate treatment of primary MEPM cells also increased expression of SRY-Box Transcription Factor 9 (SOX9), a TGFB1 target that causes cleft palate when abnormally expressed.", to acknowledge that both up- or down-regulation of SOX9 can cause cleft palate.

Please refrain from using acronyms when possible, for example "OFC" could be replaced with "gene associated with oral or facial clefts. This would make the text more accessible to the general readership of this journal.

- We have changed 9 occurrences of "OFC genes" with the suggested "genes associated with orofacial clefts". The only other acronyms used extensively are

MEPM and HEPM, which we believe make the text less cumbersome to read. Most other acronyms are in the methods section, which can be helpful for technical reasons.

Cell cycle and apoptosis genes were also identified in the IPA. Increased apoptosis and decreased cell proliferation could also cause clefting. The focus on TGF-beta is great, but the authors also could have focused on a direct mechanism by looking at cell proliferation and apoptosis.

- Respectfully, we argue that cell proliferation and apoptosis are not necessarily direct mechanisms – they are frequently downstream consequences. Our identification of topiramate upregulation of TGFB1 protein level via GABA receptor signaling offers a novel and distinct upstream etiology that now needs to be tested *in vivo*. There is a strong possibility that cell proliferation and apoptosis may be involved downstream.

This is interesting: "We saw a significant increase in intracellular TGFb1 level at the physiological 50μM topiramate treatment (Figure 2E)." We don't expect TGFb1 to function intracellularly. Was there not an increase in TGFb1 in the media, extracellular portion? Or did the authors not check? IF the authors have not checked, I suggest checking and comparing TGF-beta in the media as well.

- We showed increase in TGFB1 protein both intracellularly (Fig.2A-E) and in Western blots of total lysates (Fig2F-G). We agree with the reviewer that we don't expect TGFB1 to function intracellularly. Thus, the fact that we show P-SMAD2 upregulation, means that TGFB1 is likely secreted and working extracellularly, as expected.

Figure 2A-E: The representative cells for 250 uM topiramate show a clear increase in fluorescence. However, D, 1mM topiramate looks like it has less fluorescence than C or B. If there is indeed a greater fluorescence in most cells treated with 1mM topiramate, please find a different representative image. The apparent discrepancy between fluorescence in figure 2D and the quantification in E made me wonder about how the quantification of fluorescence was measured. There are no details about whether the fluorescence is quantified and normalized to the number of cells or if each individual cell is outlined as a region of interest. If the fluorescence is not normalized to the number of cells in an image in any way, the fluorescence quantification is questionable. These details should be provided to allow the reader to interpret the analysis.

- We have now replaced the images in Figure 2A-D with those more representative of the composite analysis in 2E. Individual cells were imaged, and the levels of TGFB1 fluorescence were quantitated in at least 30 cells per treatment from 3 independent experiments using NIH ImageJ software. Briefly, we used NIH ImageJ to calculate the corrected total cell fluorescence (CTCF) in each cell, using the formula: CTCF = Integrated Density – (Area of selected cell x Mean fluorescence of background readings). This information is now included in the methods section.

I would be interested to see if the upregulation of TGF-beta1 is due to an increase in transcription of TGF-beta1 on the mRNA level or only at the level of protein. Please perform Q-RT PCR to determine if mRNA levels of the ligand are upregulated

- Thank you for the reminder. We had previously performed qRT-PCR on HEPM cells treated with topiramate and observed no change in transcript level of *TGFB1*. We have now repeated the analysis on MEPM cells treated with topiramate and show that there is no significant change in transcript level of *Tgfb1*. We have included both pieces of data as a new **Supplementary Figure 1**. These qRT-PCRs showing no change in mRNA transcripts were carried out at the end of the 6-hour topiramate treatment. qRT-PCR analysis at different timepoints during the length of treatment needs to be done to reveal the exact temporal mRNA dynamics upstream of TGFB1 protein changes, however, these experiments are beyond the scope of the present studies.

Were P-Smad2/3 levels from the nuclear fraction or total cell lysates? Also, were Smad2 protein levels also quantified? It would be interesting to know if Smads are also upregulated or if only the phosphorylation of Smad2/3 are increased with the topiramate treatment.

- We have now included western blot analysis of total SMAD2 and show that there is no significant change in its levels.

Consider changing color scheme from Red and Green in Figure 4 to colors that are colorblind friendly.

- Thank you for pointing out this easy yet highly important fix. We have changed the color scheme in both figures 1 and 4, and will do so in all our future publications moving forward.

Additional Editor Comments:

The reviewers suggest some important revisions to the text, which should be addressed. Also, several experiments are suggested. Validating the effects of the pathways would involve several experiments. Although these are good suggestions, the scientific contribution without these experiments is reasonable. The other reviewer suggests some quantitative PCR to measure TGF-beta mRNA levels and measurement of SMAD2 protein levels for comparison to p- SMAD2 levels. I recommend these experiments to improve the scientific completeness of the study.

- We have now included the qRT-PCR analysis of TGF-beta mRNA in both HEPM and MEPM cells following topiramate treatment as a new supplemental figure (S1 Fig).
- We have also included total SMAD2 western blot analysis to Fig 2.

Journal Requirements:
When submitting your revision, we need you to address these additional requirements.

1. Please ensure that your manuscript meets PLOS ONE's style requirements, including those for file naming. The PLOS ONE style templates can be found at heps://journals.plos.org/plosone/s/file?id=wjVg/PLOSOne_formatting_sample_main_body.pdf and heps://journals.plos.org/plosone/s/file?id=ba62/PLOSOne_formatting_sample_title_authors_affiliations.pdf

Done.

2. Please provide the product number and any lot numbers of the antibodies used for Western blotting.

Done.

3. At this time, we ask that you please provide scale bars on the microscopy images presented in Figure 2 and refer to the scale bar in the corresponding Figure legend.

Done.

4. Please provide additional information about each of the cell lines used in this work, including any quality control testing procedures (authentication, characterisation, and mycoplasma testing). For more information, please see hep://journals.plos.org/plosone/s/submission-guidelines#loc-cell-lines.

Done.

5. At this time, we request that you please report additional details in your Methods section regarding animal care, as per our editorial guidelines: 1) Please provide details of the number of animals used in this study, and the source of the mice, 2) please provide details of animal welfare (e.g., shelter, food, water, environmental enrichment), and 3) please describe the age of the embryos and the method of sacrifice. Thank you for your aeention to these requests.

Done.

6. PLOS ONE now requires that authors provide the original uncropped and unadjusted images underlying all blot or gel results reported in a submission's figures or Supporting Information files. This policy and the journal's other requirements for blot/gel reporting and figure preparation are described in detail at heps://journals.plos.org/plosone/s/figures#loc-blot-and-gel-reporting-requirements and heps://journals.plos.org/plosone/s/figures#loc-preparing-figures-from-image-files. When you submit your revised manuscript, please ensure that your figures adhere fully to

these guidelines and provide the original underlying images for all blot or gel data reported in your submission. See the following link for instructions on providing the

original image data: heps://journals.plos.org/plosone/s/figures#loc-original-images-for-blots-and-gels.

Done. We have now included a Supplementary file with raw images (S1 raw images).
